# 3D-Analysis of the Proximal Humeral Anatomy before and after Stemless Shoulder Arthroplasty—A Prospective Case Series Study

**DOI:** 10.3390/jcm10020259

**Published:** 2021-01-12

**Authors:** Matthias Koch, Borys Frankewycz, Andreas Voss, Max Kaeaeb, Sebastian Herrmann, Volker Alt, Stefan Greiner

**Affiliations:** 1 Department of Trauma Surgery, University Medical Centre Regensburg, 93053 Regensburg, Germany; matthias.koch@ukr.de (M.K.); borys.frankewycz@ukr.de (B.F.); andreas.voss@ukr.de (A.V.); kaeaeb@sporthopaedicum.de (M.K.); volker.alt@ukr.de (V.A.); 2 Sporthopaedicum Regensburg/Straubing, 93053 Regensburg, Germany; 3 Helios Klinik Emil von Behring, 14165 Berlin, Germany; sebastian.herrmann@helios-gesundheit.de

**Keywords:** stemless shoulder arthroplasty, anatomical shoulder arthroplasty, osteoarthritis, 3D-evaluation, shoulder

## Abstract

Background: Stemless shoulder arthroplasty (SSA) is used to anatomically reconstruct proximal humerus geometry and preserve proximal humerus bone stock. The current literature lacks 3D-analysis of pre- and postoperative proximal humeral anatomy after SSA. The aim of this study was to prospectively analyze the humeral head anatomy using a computer-assisted topography mapping technique after SSA in relation to the preoperative status and the contralateral (not affected) side. Methods: Twenty-nine patients (mean age: 63.5 ± 11.7 years) affected by primary shoulder osteoarthritis and treated with SSA were included. Preoperative and postoperative CT scans of the affected and contralateral sites were analyzed regarding joint geometry. Clinical outcome was assessed by Constant and Disabilities of the Arm, Shoulder and Hand (DASH) score shortly before and one year after surgery. Results: Clinical outcome improved significantly. No correlation between clinical outcome and the evaluated anatomical parameters was found. There was a significant decrease of the humeral head height (*p* < 0.01) and radius (*p* = 0.03) in the preoperative versus the postoperative joint geometry. The comparison to the contralateral site showed also a significant decrease of the humeral head height (*p* < 0.01). All other parameters showed no significant differences. Conclusion: Proximal humeral anatomy can be almost anatomically reconstructed by SSA. Solely the humeral head height differs significantly to the preoperative as well as contralateral morphology.

## 1. Introduction

Shoulder arthroplasty was initially introduced in the 1950s by Charles Neer for the treatment of proximal humerus fractures [1,2]. Since then, the indication for shoulder arthroplasty was widened for shoulder osteoarthritis and many techniques and designs were developed to anatomically reconstruct the joint morphology and to achieve almost normal joint function. During this process, many stem-related complications including intraoperative humeral fracture, loosening, stress shielding, or periprosthetic humeral fracture were described [3]. Consequently, stem designs were revised regarding bony anchoring techniques and gradually shortening of the humeral stem implants. In 2004, the first stemless shoulder arthroplasty implant was introduced by Biomet in Europe [2,4] and, since then, many other stemless shoulder endoprostheses were developed [5]. The advantages of this type of prosthesis are obvious: the elimination of the humeral stem and the isolated metaphyseal fixation allow for a more flexible reconstruction of the anatomical morphology in degeneratively changed and deformed shoulder joints. Furthermore, the risk of periprosthetic fractures is significantly reduced. It also offers many additional opportunities in revision surgery due to the preserved bone stock [1,2,3,4]. According to the current literature, many studies describe a promising clinical outcome and radiologic results on X-ray analysis [1,6,7,8]. However, just a few studies investigate the shoulder joint geometry after stemless shoulder arthroplasty. All of the available studies evaluate joint morphology based on two dimensional X-ray analysis and the comparison of the preoperative and postoperative joint status [9,10,11,12]. The current literature lacks any data concerning three-dimensional computer-tomography-based joint geometry analysis after stemless shoulder arthroplasty.

Thus, the aim of the present study was to prospectively analyze the humeral head anatomy using a computer-assisted topography mapping technique in patients after stemless shoulder arthroplasty in relation to the preoperative osteoarthritic and the not affected contralateral side as an assumed original healthy anatomy. Furthermore, clinical outcome was correlated to the accuracy of the reconstructed humeral head geometry.

## 2. Experimental Section

### 2.1. Study Design

A prospective study design was chosen to evaluate a case series of patients who were scheduled to undergo stemless shoulder arthroplasty due to primary shoulder osteoarthritis after unsuccessful non-operative therapy in the period of September 2010–June 2012. The study was approved by the institutional review board (code: (feki) 012/1399). Written consent for participation was obtained from all participants before inclusion.

### 2.2. Patient Selection

According to the inclusion criteria, a total of *n* = 29 patients, aged between 42 and 94 years, were selected for inclusion in the present study. Inclusion criteria were defined as previous unsuccessful conservative therapy as well as a contralateral shoulder without previous injuries, other than age-related degenerative changes or surgical interventions, in order to serve as a control. Patients with incomplete data samples or withdrawn consent forms were excluded.

### 2.3. Subjective and Functional Outcome Scoring

Functional and subjective outcomes were evaluated prior to surgery and 12 months after surgery using the Disabilities of the Arm, Shoulder and Hand (DASH) score [13] and the Constant score (CS) [14].

### 2.4. Radiological Analysis

A three-dimensional evaluation CT scan of the affected and the contralateral shoulder joint, as well as the epicondylar axis of the distal humerus, was performed prior to stemless shoulder arthroplasty. After surgery, an additional postoperative CT scan of the operated shoulder was performed. Based on these CT scans, the following evaluation and comparison were accomplished by the IVS Technology GmbH (Chemnitz, Germany) using the VoXim^®^ 6.3 Modul Image Fusion software. The following geometrical parameters were analyzed: humeral head retroversion, inclination, medial and posterior offset, defined as surgeon-dependent parameters. Predetermined parameters (prosthesis-dependent) were also analyzed: humeral head size (height and radius), as well as the minimal and maximal resection diameter.

**Figure 1:**
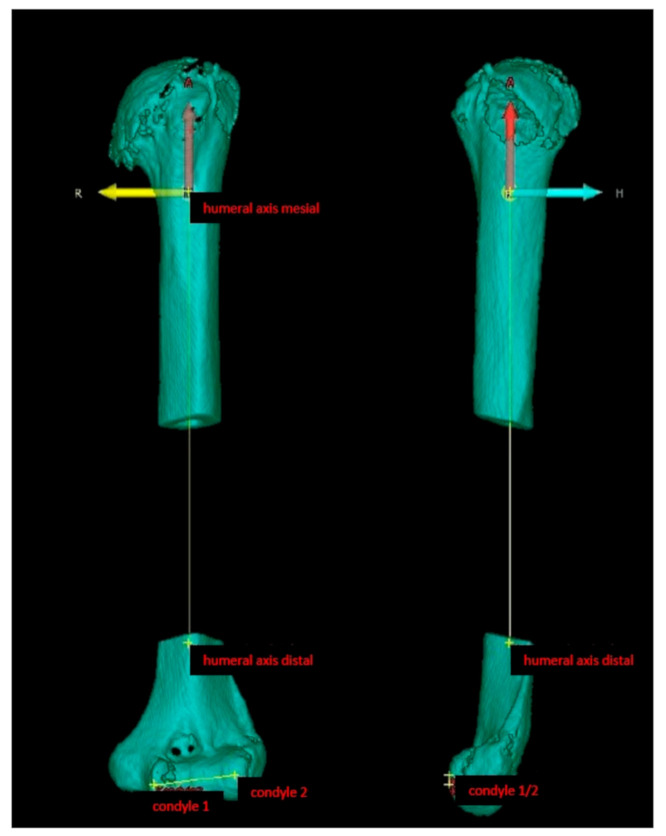
Creation of a humeral coordinate system with the condyle–humeral axis as a standardized reference.

For calculation of the morphologic parameters, a technique established in orodental research and also already used for evaluation of reproducibility of anatomical landmarks and the accuracy of different types of computer tomography was used [15]. In particular, the humerus scans were set in a coordinate system, which was virtually overlayed over the scans for software-based assessment. All required calculation points were defined by IVS Technology GmbH and independently double-checked and verified by one project collaborator (SH) and the senior author. First, the center of the condyle–humeral axis and the distal and proximal humeral center were set as virtual references for a humeral coordinate system (Figure 1).

**Figure 2:**
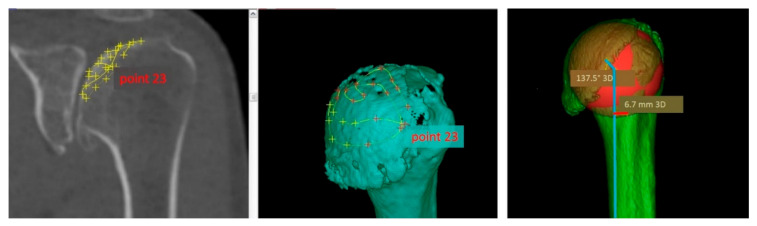
Scatter diagram of the articular humeral head surface.

Secondly, the articular surface of the humeral head was plotted based on a scatter diagram of 24 different localized points on the three-dimensional CT reconstruction (Figure 2).

**Figure 3:**
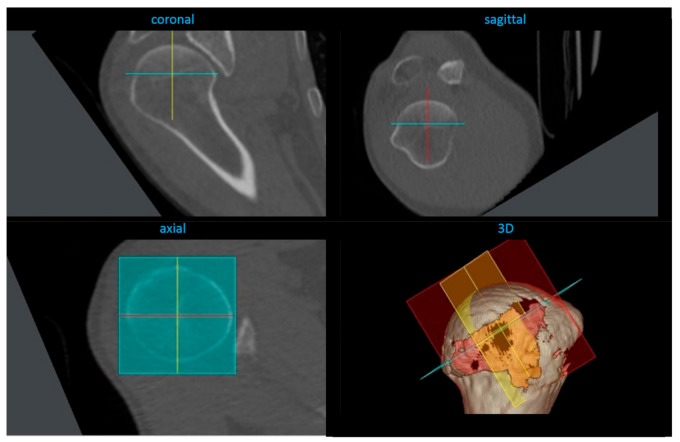
Three-dimensional humeral head coordinate system in sagittal, axial, and coronal planes and a 3D reconstruction.

Based on these marks, the software created a three-dimensional humeral head coordinate system (Figure 3).

**Figure 4:**
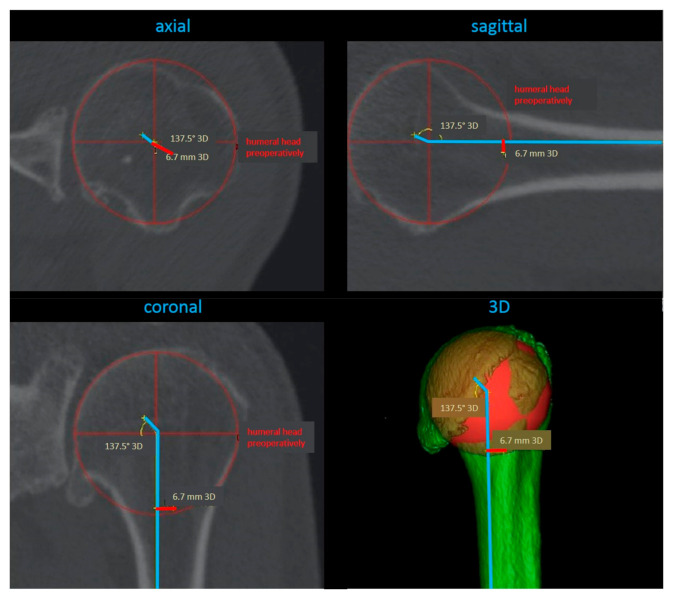
Calculation of the geometrical parameters in sagittal, axial, and coronal planes, respectively, and a 3D reconstruction.

The distance from the humeral head center and condyle–humeral-axis center served as a scale for calculations of the geometrical parameters. These were calculated within the three-dimensional humeral head coordinate system in the sagittal, axial, and coronal planes, respectively (Figure 4).

### 2.5. Surgical Procedure

All surgeries were performed using the same approach, technique, and prosthesis (Affinis Short, Mathys, Bettlach, Switzerland) by the senior author. In all patients, the stemless shoulder arthroplasty was performed using a delto-pectoral approach. After inspection of the rotator cuff and localization of the biceps tendon, the bicipital groove was exposed. After marking the subscapularis tendon with size 2 FiberWire^®^ sutures (Arthrex, Munich, Germany), the minor tuberosity was osteotomized and mobilized. After tenotomy of the long head of the biceps tendon, the humeral head was exposed and resected using the manufacturer’s humeral head resection device. For glenoid preparation, a guide pin was inserted with the manufacturer’s alignment device and the glenoid surface was reamed with a cannulated reamer. Reaming was performed in accordance with the preoperative planning. The peg holes were drilled and temporarily filled with a sponge in order to keep them dry and clean for cementing. Cement was filled in the peg holes retrogradely. The two-pegged polyethylene glenoid component was pressed into the prepared glenoid using an impactor. Manual pressure was maintained until the cement was hardened. Humeral metaphysis preparation was performed according to the manufacturer’s instructions. The humeral stem was press-fit implanted after assessing the humeral head diameter in situ and according to the preoperative planning. Implant head size was chosen according to the minimal resection diameter of the metaphysis rather than the resected humeral head.

The subscapularis tendon, attached to the minor tuberosity, was re-fixated transosseously using number 2 FiberWire^®^ sutures. All patients underwent a standardized rehabilitation program that consisted of a three-week immobilization phase with immediate passive exercises and active mobilization after six weeks.

### 2.6. Statistical Analysis

Statistical analysis was performed using SAS software (SAS Institute, Cary, NC, USA) to determine relationships between variables. Data are presented as mean ± standard deviation (SD) and range [minimum (min)–maximum (max)]. For assessing Gaussian distribution, the Shapiro Wilks test was conducted. For comparison of normal distributed data, the paired *t*-test was used. For non-normal distributed data, the Wilcoxon test was used. Correlations between variables were evaluated using a Pearson correlation test. A probability value (*p*) of ≤0.05 was considered to be significant for each test, respectively.

## 3. Results

### 3.1. General Data

A total of 29 patients were included in this study. The mean age of the patients at the time of surgery was 63.5 ± 11.7 years [range: 42–94; 25% percentile: 55 years; median: 65 years; 75% percentile: 70 years]. Overall, 9 male and 20 female patients participated in the study. In 12 cases, surgery was performed on the right shoulder; in 17 cases, on the left shoulder.

### 3.2. Clinical Outcome

The mean CS increased significantly from 34.9 ± 14.3 points preoperatively to 73.7 ± 13.6 points (*p* < 0.01) one year postoperatively. The mean DASH score significantly decreased one year after stemless shoulder arthroplasty from 47.4 ± 16.7 points to 11.8 ± 12.1 points (*p* < 0.01). Overall, none of the evaluated geometrical parameters (humeral head retroversion, inclination, medial and posterior offset, humeral head size (height and radius), and minimal and maximal resection diameter) showed any correlation with the patient-related outcome measures (CS and DASH).

### 3.3. Radiological Analysis

Prosthesis-dependent parameters (humeral head height and radius) were significantly different after stemless shoulder arthroplasty in comparison to the initial values (height: *p* < 0.01; radius: *p* = 0.03). Surgeon-depending parameters (retroversion, inclination, medial and posterior offset), as well as the minimal and maximal resection diameter, showed no significant differences. Regarding the contralateral side, preoperatively proximal humeral retroversion (*p* < 0.01) and humeral head radius (*p* < 0.01), as well as minimal (*p* = 0.05) and maximal (*p* = 0.03) resection diameter, differed significantly. After stemless shoulder arthroplasty, retroversion (*p* < 0.01) and humeral head height (*p* < 0.01) were significantly different in comparison to the contralateral shoulder (see Table 1).

## 4. Discussion

This study analyzed the joint geometry after anatomical stemless shoulder arthroplasty on a three-dimensional plotting grid from CT-scan-derived data. To the best of our knowledge, this is the first study that investigated pre- and postoperative findings of stemless shoulder arthroplasty three-dimensionally. The most important finding of this study was that stemless shoulder arthroplasty enables orthopedic surgeons to anatomically reconstruct the individual proximal humeral geometry. Almost all parameters showed no significant differences when comparing the intra-individual pre- and postoperative joint geometry and are also in accordance to described parameters in the literature [16,17,18,19,20,21]. This is particularly relevant since there is a very high variability of proximal humeral anatomy [19]. Investigation of the proximal humeral geometry on human cadavers for the development of prosthetic designs began in the 1990s [16,17,20]. Since then, many features were modified to pay regard to individual shoulder biomechanics and joint kinematics. Modular systems were introduced to fit anatomical variations [2,17]. A significant development was the introduction of stemless designs. The reduction of the length of the humeral stem was aimed to avoid stem-related complications and to preserve bone stock integrity for potential future revision surgeries, as well as to achieve a more modifiable humeral implantation of shoulder prosthesis according to the individual anatomical situation [10]. Stemless shoulder arthroplasty shows promising results in the short- and mid-term follow up [22]. However, only a few studies have investigated shoulder joint geometry after anatomical stemless shoulder arthroplasty [9,10]. Von Engelhardt et al. evaluated the restoration of joint geometry and clinical outcome after stemless shoulder arthroplasty in 21 patients and Kadum et al. analyzed joint geometry of 70 stemless shoulder prostheses. Both authors reported a reliable reconstruction of the individual joint anatomy and a significant improvement of the Constant and DASH scores. However, in both studies, joint morphology was assessed by two-dimensional plane X-rays only. A major limitation of 2D analysis is the solely projective character of imaging and the omittance of geometrical data in the third plane. Three-dimensional CT measurements are more reproducible in proximal humerus joint morphology assessment than plain radiography [23] and are indispensable for full anatomical evaluation.

Another important aspect of this study was the comparison of the pre- and postoperative joint geometry to the contralateral healthy site. The significant differences in the humeral head radius and minimal and maximal resection diameters to the preoperative and contralateral joint status might be explained by osteoarthritic changes of the affected shoulder joint, which are removed as part of osteophyte resection during shoulder replacement. Interestingly, the humeral head height of the operated shoulder differed significantly compared to the preoperative status and also the contralateral side. This study shows that anatomical shoulder arthroplasty does not completely reconstruct humeral head height to the extent of the “healthy” contralateral or preoperatively degenerative status. One reason for this might be that the humeral head is not part of a sphere but has two different diameters and cannot be completely anatomically reconstructed with available implants. However, the relevance of this is ambiguous. Since none of the evaluated geometrical parameters showed a significant correlation to the patient-related outcome measures (Constant score and DASH score) and all patients have shown improving clinical results, the reduced humeral head height seems not to be detrimental for the shoulder function regarding the short-term results. Like in the former studies [9,10], both the Constant and DASH scores improved significantly within one year. However, data investigating long-term results are sparse [22]. Nothing is known concerning a correlation of the patient-related outcome measures and geometrical parameters in the long term. In order to evaluate long-term results and especially potential long-term failures of stemless shoulder prostheses, the geometrical features assessed in this study will be of value for further investigations.

Despite the unique data collection, the present study has some limitations which should be considered in the interpretation of its results. In the present study, surgery was performed using solely one type of stemless shoulder prosthesis. Thus, any generalization to other types of stemless prostheses may not be applicable. However, considering the total number of patients, the inclusion of different types of stemless prostheses would be a relevant bias in this study. Even though the sample size of 29 patients is quite small, it is still representative to determine values of anatomical landmarks sufficiently, especially considering radioprotection restrictions. Multiple CT scans of the affected and contralateral side expose the patients to a not insignificant amount of radiation loads; therefore, the minimized sample size can be justified. One further limitation is the range of age of the investigated patients. According to the current literature, there are age-related differences regarding proximal humeral bone structure and anatomy. However, the intra-individual comparison of the preoperative, postoperative, and contralateral anatomy should account for this limitation. Regarding the clinical outcome, a one-year follow up has to be considered as limitative. However, showing significant improvement of clinical outcome is in accordance with the available data in the current literature, supporting the good midterm results of stemless shoulder arthroplasty. One further aspect is the isolated analysis of the proximal humeral anatomy without evaluation of the corresponding glenoid as its immediate and essential joint partner regarding functional outcome. Despite all limitations, the data retrieved from this study provide a basis for future biomechanical and prospective long-term studies.

## 5. Conclusions

The present study shows that stemless shoulder arthroplasty is able to reconstruct proximal humeral geometry almost anatomically. However, comparing postsurgical joint morphology to the preoperative and contralateral status, a significantly reduced humeral head height was detected. Clinical outcome improved significantly after one year and did not correlate with any geometrical parameter. Further studies with a larger population and long-term evaluation are required to investigate the clinical impact of these findings.

## Figures and Tables

**Table 1:** Geometrical parameters of humeral joint anatomy.

Parameter		Mean	SD	Min	Max
retroversion	preop.	20.9 *	13.8	1.9	52.8
	postop.	21.8 *	16.4	1.5	51.0
	contralateral	29.6 *	12.5	8.7	52.8
inclination	preop.	125.5	17.2	41.0	142.7
	postop.	130.3	7.9	114.4	146.2
	contralateral	131.3	5.9	119.3	141.6
medial offset	preop.	4.0	1.5	0.6	6.4
	postop.	3.6	2.2	0.3	10.2
	contralateral	4.1	1.8	0.2	6.9
posterior offset	preop.	2.3	1.5	0	5.1
	postop.	1.6	1.5	0	5.5
	contralateral	1.6	1.3	0.1	5.6
head height	preop.	17.1 *	3.1	10.6	23.8
	postop.	14.5 *	1.4	13.0	18.0
	contralateral	16.9 *	2.5	12.8	22.2
head radius	preop.	24.4 *	3.0	18.7	31.6
	postop.	23.5	1.4	22.0	27.0
	contralateral	23.4 *	2.8	18.6	29.1
minimal resection diameter	preop.	46.9 *	5.8	37.2	58.4
	contralateral	44.8 *	4.8	37.6	57.9
maximal resection diameter	preop.	50.4 *	6.1	39.6	63.9
	contralateral	47.9 *	5.1	38	60.6

* = indicates significant differences. SD = standard deviation.
